# Two Decades of Global Progress in Authorized Advanced Therapy Medicinal Products: An Emerging Revolution in Therapeutic Strategies

**DOI:** 10.3389/fcell.2020.547653

**Published:** 2020-12-17

**Authors:** Roya Ramezankhani, Shukoofeh Torabi, Neda Minaei, Hoda Madani, Siamak Rezaeiani, Seyedeh Nafiseh Hassani, Adrian P. Gee, Massimo Dominici, Daniela Nascimento Silva, Hossein Baharvand, Ensiyeh Hajizadeh-Saffar

**Affiliations:** ^1^Department of Regenerative Medicine, Cell Science Research Center, Royan Institute for Stem Cell Biology and Technology, Academic Center for Education, Culture and Research, Tehran, Iran; ^2^Department of Applied Cell Sciences, Faculty of Basic Sciences and Advanced Medical Technologies, Royan Institute, Academic Center for Education, Culture and Research, Tehran, Iran; ^3^Department of Stem Cells and Developmental Biology, Cell Science Research Center, Royan Institute for Stem Cell Biology and Technology, Academic Center for Education, Culture and Research, Tehran, Iran; ^4^Advanced Therapy Medicinal Product Technology Development Center, Royan Institute for Stem Cell Biology and Technology, Academic Center for Education, Culture and Research, Tehran, Iran; ^5^Division of Hematology and Oncology, Department of Pediatrics, Baylor College of Medicine, Houston, TX, United States; ^6^Division of Oncology, Department of Medical and Surgical Sciences for Children & Adults, University of Modena and Reggio Emilia, Modena, Italy; ^7^Karolinska Institutet, Stockholm, Sweden; ^8^Health Institute of Technology, SENAI-CIMATEC, Salvador, Brazil; ^9^Department of Developmental Biology, University of Science and Culture, Tehran, Iran

**Keywords:** advanced therapy medicinal products, ATMP, safety, efficacy, market size

## Abstract

The introduction of advanced therapy medicinal products (ATMPs) to the global pharma market has been revolutionizing the pharmaceutical industry and has opened new routes for treating various types of cancers and incurable diseases. In the past two decades, a noticeable part of clinical practices has been devoting progressively to these products. The first step to develop such an ATMP product is to be familiar with other approved products to obtain a general view about this industry trend. The present paper depicts an overall perspective of approved ATMPs in different countries, while reflecting the degree of their success in a clinical point of view and highlighting their main safety issues and also related market size as a whole. In this regard, published articles regarding safety, efficacy, and market size of approved ATMPs were reviewed using the search engines PubMed, Scopus, and Google Scholar. For some products which the related papers were not available, data on the relevant company website were referenced. In this descriptive study, we have introduced and classified approved cell, gene, and tissue engineering-based products by different regulatory agencies, along with their characteristics, manufacturer, indication, approval date, related regulatory agency, dosage, product description, price and published data about their safety and efficacy. In addition, to gain insights about the commercial situation of each product, we have gathered accessible sale reports and market size information that pertain to some of these products.

## Introduction

Based on Directive 2001/83/EC, medicinal products in Europe have been defined as any substance or combination of substances that have the capability to treat or prevent diseases in humans or may be used with the purpose to restore, correct, or modify physiological functions in conjunction with the capability to be used for medical diagnosis in humans. With the advent of new gene and/or cell therapies and in order to assure their appropriate quality, safety, and efficacy, these therapies were introduced into the European medicinal product legislation in June 2003 as a new class of medicinal products, which were later called ATMPs. In 2007, Regulation (EC) No. 1394/2007, a specific regulation for ATMPs, was established by the EU Commission. This regulation divides ATMPs into four distinct types: GTMPs, SCTMPs, TEPs, and the combined ATMPs (cATMPs). GTMPs are products directly related to therapeutic, prophylactic, or diagnostic effects with a recombinant nucleic acid sequence. SCTMPs are products that contain substantially manipulated cells or tissues, or the cells or tissues not intended to be used for the same essential function(s) in the recipient and the donor. TEPs are engineered cells or tissues that have the properties of regenerating, repairing, or replacing human tissue, all in accordance with the medicinal products general definition and finally cATMPs comprise another type of these products and contain one or several medical devices that are an integral part of the GTMPs, SCTMPs, or TEPs (Hanna et al., 2016a; Detela and Lodge, 2019). Also, each regulatory authority may provide a certain type of definition for ATMPs. For example, in US according to FDA, advanced therapies are regulated as biologic products, similar to EU classification. Biological products consist of allergenic products that includes allergen extracts, allergen patch tests, and antigen skin tests, blood and blood products, vaccines, xenotrasplants, and ATMPs which constitutes two sub-categories: CGTs (Integra, 2019). “Cellular immunotherapies, cancer vaccines, and other types of both autologous and allogeneic cells for certain therapeutic indications, including hematopoietic stem cells and adult and embryonic stem cells that have been subject to substantial *ex vivo* manipulation constitute cellular therapy based products, while modifying the expression of a gene or changing the biological properties of living cells for therapeutic use compose human gene therapy based products” (Genzyme, 2019). Moreover, “combination products include products that are comprised of two or more regulated components, i.e., drug/device, biologic/device, drug/biologic, or drug/device/biologic.” The MFDS in South Korea also define the cell therapy product as “a medicinal product manufactured through physical, chemical, and/or biological manipulation, such as *in vitro* culture of autologous, allogeneic, or xenogeneic cells. However, this definition does not apply to a case where a medical doctor performs minimal manipulation (e.g., simple separation, washing, freezing, thawing, and other manipulations, while maintaining biological properties) that does not cause safety problems of the cells in the course of surgical operation or treatment at a medical center.” And a gene therapy product is defined as “a genetic material or a medicinal product containing such genetic material intended to be administered to human beings for treatment of disease (Choi et al., 2015). The regulatory guidelines regarding the (pre)submission, details of approval procedures, marketing authorization etc. have described thoroughly elsewhere (Detela and Lodge, 2019; EU, 2020; Luria et al., 2020). The need to establish effective therapeutic approaches to treat incurable diseases, notably, inherited genetic conditions, blood related disorders, malignancies, neurodegenerative diseases, tissue regeneration, and provide a bridge for patients awaiting organ transplantation has encouraged the increased use of ATMPs in medical sciences. Interestingly, a significant growth in the research and development phase along with the clinical use of ATMPs has been observed in recent years. In this regard, based on the results of three clinical trials databases: ClinicalTrials.gov, the International Clinical Trials Registry Platform (ICTRP) of the World Health Organization (WHO), and EudraCT, 939 clinical trials of ATMPs conducted between 1999 and June 2015 (Hanna et al., 2016b). This would indicate an increase in investment by big pharma sponsors for ATMPs (Ten Ham et al., 2018). Of note, potential challenges that exist in terms of the development of ATMPs include the specific requirements for high-technology equipment, difficulty with manufacturing processes, complicated trial design, establishment of robust assays for validation of identity and functionality, achieving an expected high efficacy, avoidance of probable long-term adverse events, regulatory considerations in terms of regulatory cost burden and timelines etc., and, in particular, financial issues that provide situations where the product cannot be sold at a sufficiently high price to establish a commercially viable product (Mount et al., 2015; Elsanhoury et al., 2017; Lee, 2018). ATMPs are based on a diverse set of most advanced technologies (Elsanhoury et al., 2017), therefore, there is an increased need for the technical/academic personnel involved directly and professionally in ATMP development (Lee, 2018). Besides, regarding the rare nature of the diseases that ATMPs are mostly developed for, there are concerns in relation with trial design such as the low number of patients, insufficient knowledge respecting the disease pathogenesis and some issues with the interpretation of endpoints for new indications (Lee, 2018). Also, the statistical analysis of safety and efficacy is affected by the limited number of participants (Viganò et al., 2018). On the other hand, validating these products particularly with regard to identity, purity, and potency is of great importance. The restricted accessible appropriate standards and reference material along with an inadequacy in certain guidelines are the other challenges in this regard (McConaghie, 2017).

Financial issues may be one of the main challenges that can negatively influence the company and consumers. A well-known example, Glybera, is a gene therapy based drug for a rare familial LPLD (European Medicines Agency, 2020b). Its marketing authorization expired on October 28, 2017 following a decision by the marketing authorization holder to not apply for a renewal. The drug was proven to be a commercial failure because a single dose treatment cost over one million euro per patient, in addition to the low market size due to the fact that LPLD is a ultra-rare disease (Senior, 2017; Cuende et al., 2018).

Cuende et al. (2018) previously described cell therapy products with market authorization (Food and Drug Administration, 2019a), in this extensive review thanks to available information in the regulatory agencies and related company’s web resources, articles, and other data sources, we in-depth dissected and classified cell, gene, and tissue engineering products (Tables 1–3). Data are presented in detailed tables that has been categorized in terms of product’s definition, manufacturer, indication, approval date and related regulatory agency, product dosage and description, price, and related references. In addition, based on clinical trials data, we have further discussed each ATMP’s safety and efficacy points, categorized by the common indication within each group. Also included is a definition of the available market sizes and sale reports for the related products in an attempt to clarify the commercial point of view for each of the GTMPs, SCTMPs, and TEPs fields.

**TABLE 1:** List of approved cell therapy medicinal products (CTMPs).

No.	Trade name/proper name	Manufacturer	Indication	Approved by/date	Product dosage	Product form	Product description	AT/AL	Price
1	Hemacord (Food and Drug Administration, 2019g) HPC, cord blood	New York Blood Center, Inc. (United States)	HSCT	US FDA 2011 November	A minimum of 5 × 10^8^ total nucleated cells with at least 1.25 × 10^6^ viable CD34 + cells at the time of cryopreservation	Bag	Human cord blood-derived HPCs	AL	NA/Generally, an average allogeneic HSCT costs US $200,000
2	HPC, cord blood (Food and Drug Administration, 2019f)	Clinimmune Labs, UCCBB (United States)	HSCT	US FDA 2012 May	A minimum dose of 2.5 × 10^7^ nucleated cells/kg at cryopreservation	Bag	Human cord blood-derived HPCs	AL	
3	Ducord HPC, cord blood (Food and Drug Administration, 2019e)	Duke University School of Medicine (United States)	HSCT	US FDA 2012 October	A minimum dose of 2.5 × 10^7^ nucleated cells/kg at cryopreservation	Bag	Human cord blood- derived HPCs	AL	
4	Allocord HPC, cord blood (Yeh et al., 2018; Food and Drug Administration, 2019h)	SSM Health Cardinal Glennon Children’s Hospital (United States)	HSCT	US FDA 2013 May	A minimum of 5 × 10^8^ TNC with at least 1.25 × 10^6^ viable CD34 + cells at cryopreservation	Bag	Human cord blood- derived HPCs	AL	
5	HPC, cord blood (RxList, 2018)	LifeSouth Community Blood Centers, Inc. (United States)	HSCT	US FDA 2013 June	2.5 × 10^7^ nucleated cells/kg	Bag	Human cord blood- derived HPCs	AL	
6	HPC, cord blood (Food and Drug Administration, 2019c)	Bloodworks (United States)	HSCT	US FDA 2016 January	Minimum dose of 2.5 × 10^7^ nucleated cells/kg at cryopreservation Each bag: 5 × 10^8^ TNC with a minimum of 1.25 × 10^6^ CD34 + cells in 25 ml	Bag	Human cord blood- derived HPCs	AL	
7	Celevecord HPC, cord blood (Food and Drug Administration, 2019d)	Cleveland Cord Blood Center (United States)	HSCT	US FDA 2016 September	A minimum dose of 2.5 × 10^7^ nucleated cells/kg at cryopreservation	Bag	Human cord blood- derived HPCs	AL	
8	HPC, cord blood (Food and Drug Administration, 2019b)	MD Anderson Cord Blood Bank (United States)	HSCT	US FDA 2018 June	2.5 × 10^7^ nucleated cells/kg	Bag	Human cord blood- derived HPCs	AL	
9	Azficel-T laViv (Fibrocell Science Inc., 2013; S-Biomedics Ltd., 2019a)	Fibrocell Technologies, Inc. (United States)	Moderate to severe NLF wrinkles	US FDA 2011 June	∼18 × 10^6^ autologous fibroblasts in 1.2 ml suspension/three treatment sessions spaced at intervals of 3 to 6 weeks	Vial	Human fibroblasts	AT	$19,900 for a patient’s full course of treatment
10	Provenge Sipuleucel-T (Timmerman, 2010; Food and Drug Administration, 2019j)	Dendreon, Corp. (United States)	Asymptomatic or minimally symptomatic metastatic castrate resistant (hormone refractory) PCA	US FDA 2010 April	Minimum of 50 million activated CD54 + cells, suspended in 250 mL of Lactated Ringer’s solution	Bag	PBMNCs (primarily DCs) activated with PAP and GM-CSF	AT	$93,000 for 3 infusions
				EMA 2010 September					
11	Prochymal BM-MSCs (Rattue, 2012; Mills, 2019)	Mesoblast, Ltd., International (Australia)	Acute and refractory GvHD	US FDA 2015 June	Intravenous administration: Low (2 million cells/kg) High (8 million cells/kg)	Bag	Human BM-MSCs	AL	$200,000 per treatment
				Health Canada 2012 May					
12	Alofisell (Cx601) Darvadstrocel (Cho et al., 2015; NICE, 2019; Specialist pharmecy service, 2020)	TiGenix (United States) and Takeda (United Kingdom)	Complex perianal fistulas in CD	EMA 2018 March	5 million MSCs/ml suspension Treatment: 4 vials	Vial	Human adipose tissue- derived MSCs	AL	∼ $47,485 per treatment
13	KeraHeal (Biosolution Ltd., 2019a, b; Medical observer, 2019)	Biosolution, Co., Ltd. (South Korea)	Deep 2^*nd*^ degree burn (> 30% of the TBSA) and 3^*rd*^ degree burn (>10% of the TBSA)	South Korea MFDS 2006 May	1 ml skin-derived keratinocytes suspension/100–400 cm^2^	Vial	Human skin-derived keratinocytes	AT	∼ $3,561/100–400 cm^2^ per vial
14	Queencell (Anterogen Co., 2020; Ministry of Food and Drug Safety, 2020)	Anterogen (South Korea)	Subcutaneous tissue defects	South Korea MFDS 2010 March	Minimally manipulated ADC ≥1.0 × 10^6^/vial (1 mL). Cell volume: according to size of the subcutaneous fat defect site.	Vial	Human adipose tissue-derived adipose cell	AT	NA
15	CureSkin (Sang-jun, 2010; Biomedic, 2019; S-Biomedics Ltd., 2019b)	S. Biomedics (South Korea)	Depressed acne scars	South Korea MFDS 2010 May	50 to 100 μl (2.0 × 10^7^ cells/ml) of dermal fibroblasts per intradermal injection three times biweekly injection	Vial	Human dermal fibroblasts	AT	A 500-won coin-sized scar: ∼ $6,300 and the whole face: $11,700
16	KeraHeal-Allo (Joo-sung, 2016; Biosolution Ltd., 2019c)	Biosolution, Co., Ltd. (South Korea)	Deep 2^*nd*^ degree burns	South Korea MFDS 2015 October	One syringe (2.0 × 10^7^ skin-derived keratinocytes/1.5 ml) to the image area of 100 cm^2^	Pre-filled syringe	Human skin-derived keratinocytes suspended in a thermosensitive hydrogel	AL	∼ $628 per 1.5-ml
17	Rosmir (Doo-hyun, 2018; Tego Science, 2020a, b)	Tego Sciences (South Korea)	Nasojugal groove	South Korea MFDS 2017 December	2 × 10^7^ fibroblasts cells per packaging unit; single intradermal administration	Vial	Human fibroblasts	AT	More than ∼ $ 81.38 per injection
18	Chondron (Naver, 2019; Sewon Cellontech Ltd., 2019a)	Sewon Cellontech, Corp. (South Korea)	Focal knee cartilage defect	South Korea MFDS 2001 January	1 to 6 vials per patient >12 million cultured chondrocytes per vial	Vial	Human chondrocytes	AT	∼$5,890 per treatment
19	RMS- Ossron (Sewon Cellontech Ltd., 2019b)	Sewon Cellontech, Co., Ltd. (South Korea)	Bone defects	South Korea MFDS 2009 August	1 to 6 vials containing > 12 million cultured osteoblasts per vial (0.4 ml)	Vial	Human osteoblasts	AT	NA
20	Cartistem (Cade Hildreth, 2018; Medipost, 2019; SCT, 2019)	Medipost (South Korea)	Knee osteoarthritis (ICRS grade IV)	South Korea MFDS 2012 January	500 μL/cm^2^ depending on the lesion (7.5 × 10^6^ cells in 1.5 ml)	Vial	Human umbilical cord blood-derived MSCs	AL	$19,000–21,000 for the standard treatment and an additional $10,000 for each extra treatment
21	CreaVax-RCC (Woo, 2007; Ministry of Food and Drug Safety, 2019a)	JW CreaGene (South Korea)	Metastatic renal cell carcinoma for which nephrectomy can be performed	South Korea MFDS 2007 May	8 doses (4 times, once every 2 weeks); each vial: 5.0 × 10^7^ dendritic cells in 2 ml	Vial	Human DCs	AT	∼ $27,000 (eight treatments)
22	Immuncell-LC (Sun-kyu, 2016; Ministry of Food and Drug Safety, 2019b)	Green Cross Cell, Corp. (South Korea)	Post-surgical recurrence of hepatocellular carcinoma	South Korea MFDS 2007 August	Each administration: 200 ml over a spot that contains 1.0 × 10^9^ ∼ 2.0 × 10^10^ activated T-cells (16 doses)	Bag	Human activated T lymphocytes	AT	>$4,500 per dose
23	Cupistem (Cade Hildreth, 2018; Anterogen Co., 2019; Ministry of Food and Drug Safety, 2019c)	Anterogen (South Korea)	Crohn’s fistula	South Korea MFDS 2012 January	Fistula diameter: (a) ≤ 1 cm (3.0 × 10^7^ AT-MSCs in 1 ml) (b) 1 < X < 2 cm (6.0 × 10^7^ AT-MSCs in 2 ml)	Vial	Human adipose tissue-derived MSCs	AT	$3,000–5,000 per treatment
24	Cellgram-AMI (Doo-hyun, 2019)	FCB Pharmicell (South Korea)	AMI	South Korea MFDS 2011 July	(a) Under 60 kg = 10 mL/5.0 × 10^7^ BM-MSCs (b) 61 ∼ 80 kg = 14 mL/7.0 × 10^7^ BM-MSCs c. Over 81 kg = 18 mL/9 × 10^7^ BM-MSCs	Pre-filled syringe	Human BM- MSCs	AT	$15,000 for one shot
25	Neuronata-R (Han-soo, 2018; Corestem Inc., 2019)	Corestem (South Korea)	ALS (Lou Gehrig’s Disease)	South Korea MFDS 2014 July	(0.1 ml/kg) 1.0 × 10^6^ BM-MSCs in 4 ml self-cerebrospinal fluid Twice every 4 weeks	Pre-filled syringe	Human BM-MSCs	AT	∼$55,000 annually (24 treatments)
26	Cartigrow (Das, 2018; FAQ, 2019)	Regrow Biosciences, Pvt. Ltd. (India)	Knee/ankle cartilage loss	India DCGI 2017 April	48 million chondrocytes	Vial	Human chondrocytes	AT	$1,988 per treatment
27	Ossgrow (Das, 2018; Regrow Biosciences Pvt. Ltd., 2019)	Regrow Biosciences, Pvt. Ltd. (India)	Early-stage AVN of hip	India DCGI 2017 April	1.2 × 10^7^ autologous bone cells/0.4 ml	Vial	Human osteoblasts	AT	$1,988 per treatment
28	Apceden AMDDC (Apac Biotech, 2019; Safer, 2019)	APAC Biotech (India)	Prostate, ovarian, colorectal and NSCLC	India DCGI 2017 March	6 doses (4–5 million mature DCs per dose) in 14 weeks	Vial	Monocyte-derived mature DCs	AT	$7,100–9,940 per treatment
29	Stempeucel (Jayaraman, 2016; Stempeutics Research Pvt Ltd., 2019)	Stempeutics Research (India)	CLI due to thromboangiitis obliterans (Buerger’s disease)	India DCGI 2016 May	Intramuscular injection of 1 or 2 million cells/kg body weight	Vial	Human BM-MSCs	AL	$2200 per treatment
30	Chondrocytes-T-Ortho-ACI Cartogen (Orthocell, 2018, 2019)	Orthocell (Australia)	Cartilage damage (chondromalacia patella or OCD) 18–55 years	Australia TGA 2017 March	2–5 million cells suspended in 1.0 ml of assembly medium	Bag	Human chondrocytes	AT	$4,500 per treatment
31	Temcell HS (StreetInsider, 2015; Jcr Pharmaceuticals Co., 2020)	JCR Pharmaceuticals (Japan)	Acute and refractory GvHD	Japan PMDA 2015 September	Intravenous infusion of 2 million cells/kg (each bag contains 72 million cells in 18 ml of saline) 4 ml per minute twice weekly at an interval of 3 days or more for 4 weeks	Bag	Human BM-MSCs	AL	$7,600 per bag
32	RenuDermcell (CellTech, 2019)	Cell Tech Pharmed (Iran)	Facial wrinkles and acne scars, atrophic skin lesions following skin trauma	Iran FDA 2018 January	Intradermal injection of a minimum of 3.0 × 10^7^ cells. Usually three repeated times	Vial	Human dermal fibroblasts	AT	NA
33	MesestroCell (CellTech, 2019)	Cell Tech Pharmed (Iran)	OA and knee joint arthritis	Iran FDA 2018 January	A minimum intra-articular injection of 2.0 × 10^7^ cells/knee, totally 4.0 × 10^7^ cells for both knees	Vial	BM-MSCs	AT	NA
34	RecolorCell (CellTech, 2019)	Cell Tech Pharmed (Iran)	Different types of vitiligo: focal; segmental generalized	Iran FDA 2019 February	Approximately 70,000 cells/cm^2^ area of vitiligo patches; This product is effective for vitiligo patches < 200 cm^2^	Vial	Human keratinocytes and melanocytes	AT	NA

*ADC, adipose tissue-derived cell; AL, allogeneic; ALS, amyotrophic lateral sclerosis; AMDDC, autologous monocyte-derived mature dendritic cell; AMI, acute myocardial infarction; AT, autologous; AT-MSCs, adipose tissue-derived mesenchymal stem cell; AVN, avascular necrosis; BM-MSC, bone marrow-derived mesenchymal stem cell; CD, Crohn’s disease; CLI, critical limb ischemia; DC, dendritic cell; DCGI, Drug Controller General of India; DFU, diabetic foot ulcer; EMA, European Medicines Agency; FDA, Food and Drug Administration; GM-CSF, granulocyte macrophage colony stimulating factor; GvHD, graft-versus-host disease; HCEpC, human corneal epithelial cell; HPC, hematopoietic progenitor cell; HSCT, hematopoietic stem cell transplantation; ICRS, International Cartilage Repair Society; MFDS, Ministry of Food and Drug Safety; MSC, mesenchymal stem cell; NA, not available; NLF, nasolabial fold NSCLC, non-small cell lung carcinoma; OA, osteoarthritis; OCD, osteochondritis dissecans; PAP, prostatic acid phosphatase; Pca, prostate cancer; PBMNC, peripheral-blood mononuclear cell; PMDA, Pharmaceuticals and Medical Devices Agency; TBSA, total body surface area; TGA, Therapeutic Goods Administration; TNC, total nucleated cell; UCCBB, University of Colorado Cord Blood Bank. All prices are in USD.*

**TABLE 2:** List of approved gene therapy medicinal products (GTMPs).

No.	Trade name/proper name	Manufacturer	Indications	Approved by/date	Dosage	Product form	Description	AT/AL	Price
1	Kymriah Tisagenlecleucel (Miller, 2018; Food and Drug Administration, 2019k)	Novartis Pharmaceuticals, Corp. (United States)	Refractory B-ALL or in second or later relapse for patients up to 25 years of age	US FDA 2017 August	Intravenous administration of 0.2–5.0 × 10^6^ cells/kg for ≤ 50 kg, and 0.1–2.5 × 10^8^ cells/kg for > 50 kg B-cell ALL patients up to 25 years of age and 0.6–6.0 × 10^8^ cells for adult r/r DLBCL	Bag	CD19-targeted genetically modified T-lymphocytes	AT	$475,000 for ALL and $373,000 for DLBCL
2	Yescarta Axicabtageneciloleucel (Clarke and Berkrot, 2019; Food and Drug Administration, 2019l)	Kite Pharma, Inc. (United States)	Adult patients with r/r large B-cell lymphoma after two or more lines of systemic therapy, including DLBCL not otherwise specified, primary mediastinal large B-cell lymphoma, high grade B-cell lymphoma, and DLBCL arising from follicular lymphoma	US FDA 2017 October	2 × 10^6^ CAR-T cells/kg	Bag	CD19-targeted genetically modified T lymphocytes	AT	$373,000 per treatment
3	Zolgensma Onasemnogene abeparvovec-xioi (Food and Drug Administration, 2019m)	AveXis (United States)	Pediatric patients < 2 years of age with SMA and bi-allelic mutations in the *SMN1* gene	US FDA 2019 May	1.1 × 10^14^ to 1.4 × 10^14^ vg/kg	Vial	AAV9 vector containing functional copy of the SMN1 gene	-	$2.1 million per treatment
4	Kymriah Tisagenlecleucel (European Medicines Agency, 2019)	Novartis Pharmaceuticals, Corp. (United States)	Patients up to 25 years of age with refractory B-ALL, who are in relapse post-transplantation or in second or later relapse, and adult patients with r/r DLBCL after two or more lines of systemic therapy	EMA 2018 August	Intravenous administration of 0.2–5.0 × 10^6^ cells/kg for ≤ 50 kg, and 0.1–2.5 × 10^8^ cells/kg for > 50 kg B-cell ALL patients up to 25 years of age and 0.6–6.0 × 10^8^ cells for adult r/r DLBCL	Bag	CD19- targeted genetically modified T-lymphocytes	AT	$475,000 for ALL, and $373,000 for DLBCL
5	Yescarta Axicabtagene ciloleucel (Clarke and Berkrot, 2019; Yeskarta)	Kite Pharma, Inc. (United States)	Adult patients with r/r DLBCL and PMBCL after 2 or more lines of systemic therapy	EMA 2018 August	2 × 10^6^ CAR-T cells/kg	Bag	CD19-targeted genetically modified T- lymphocytes	AT	$373,000 per treatment
6	Imlygic Talimogene laherparepvec [Imlygic, 2017; IMLYGIC (talimogene laherparepvec) | FDA, 2020]	Amgen, Inc. (United States)	Unresectable cutaneous, subcutaneous, and nodal lesions in recurrent melanoma after initial surgery	US FDA 2015 October	An initial dose of up to 4 × 10^6^ PFU/ml, followed by subsequent doses of up to 4 ml at a concentration of 10^8^ PFU/ml	Vial	Live, attenuated HSV-1 genetically modified to express hGM-CSF	-	$65,000 per treatment
				EMA 2015 December					
7	Zalmoxis (Zalmoxis, 2016; MolMed, 2019)	Molmed S.p.A. (Italy)	Haploidentical-HSCT adult patients with high-risk hematological malignancies	EMA 2016 August Withdrawn 2019 October	1 ± 0.2 × 10^7^ cells/kg	Bag	Genetically modified T-lymphocyte with a retroviral vector encoding ΔLNGFR and HSV-TK	AL	$170,000 (Italy) $186,000 (Germany)
8	Strimvelis (Mullin, 2019; Stem Cell Research, 2019)	GlaxoSmithKline (GSK, United Kingdom)	ADA-SCID	EMA 2016 May	The recommended dose range is between 2 and 20 million CD34 + cells/kg	Bag	Transduced CD34 + cells with a retroviral vector encoding human ADA	AT	$648,000 per treatment
9	Luxturna Voretigeneparvovec-rzyl (Berkrot, 2018; Food and Drug Administration, 2019n)	Spark Therapeutics, Inc. (United States)	Biallelic RPE65 mutation-associated retinal dystrophy	US FDA 2017 December	Sub-retinal injection of 1.5 × 10^11^ AAV vector genomes in a total volume of 0.3 ml for each eye	Vial	Live, non-replicating AAV2 genetically modified to express hRPE65 gene	-	$425,000 per eye
				EMA 2018 September					
10	Gendicine (Rosen, 2012; Sibiono, 2019)	Shenzhen SiBiono Gene Tech, Co., Ltd. (China)	Late-stage HNSCC or terminal-stage non-HNSCC tumors	CFDA 2003 October	Administration of 1–4 × 10^12^ VP once every 3–7 days over a course of 3–8 weeks	Vial	Recombinant adenovirus expressing human p53	-	Up to $100,000 per dose
11	Oncorine (SunWay Biotech, 2019)	Shanghai Sunway Biotech (China)	Nasopharyngeal carcinoma	CFDA 2005 November	5 × 10^11^ VP for 5 consecutive days	Vial	Recombinant human adenovirus type 5 with E1B-55kD and E3 region deletion	-	NA
12	Kymriah Tisagenlecleucel (Novartis., 2019b)	Novartis Pharmaceuticals Canada, Inc. (Canada)	3–25 year old patients with refractory B-ALL, relapsed after allogeneic SCT or ineligible for SCT, or with second or later relapse, and adult patients with r/r large B-cell lymphoma after two or more lines of systemic therapy including DLBCL not otherwise specified, high grade B-cell lymphoma and DLBCL arising from follicular lymphoma	Health Canada 2018 September	Intravenous administration of 0.2–5.0 × 10^6^ cells/kg for ≤50 kg, and 0.1–2.5 × 10^8^ cells/kg for > 50 kg B-cell ALL patients up to 25 years of age and 0.6–6.0 × 10^8^ cells for adult relapsed or refractory diffuse large B-cell lymphoma	Bag	CD19-targeted genetically modified T-lymphocytes	AT	$475,000 per treatment
13	Neovasculgen Cambiogeneplasmid (NOVARTIS, 2019a)	Human Stem Cells Institute (Russia)	PAD, including CLI caused by atherosclerosis	MOH of the Russia Federation 2011 December	2 sequential injections (i.e., 2 vials) of 1.2 mg of pCMV- veg f165 with an interval of 14 days	Vial	Plasmid encoding the CMV-VEGF (165 aa) gene	-	∼ $6600 per treatment
14	Zynteglo (ClinicalTrials, 2020)	bluebird bio (Netherlands) B.V.	Patients up to 12 years old with beta thalassemia who require regular blood transfusions	EMA 2019 May	1.2–20 × 10^6^ cells/mL dispersion for infusion	Bag	CD34 + cells encoding βA-T87Q-globin gene	AT	$1.78 million

*AAV, adeno-associated virus; ADA-SCID, adenosine deaminase severe combined immunoeficiency; AL, allogeneic; AT, autologous; B-ALL, B-cell precursor acute lymphoblastic leukemia; CAR, chimeric antigen receptor; CFDA, China Food and Drug Administration; CLI, critical limb ischemia; DLBCL, diffuse large B-cell lymphoma; EMA, European Medicines Agency; FDA, Food and Drug Administration; GM-CSF, granulocyte macrophage colony-stimulating factor; HNSCC, head and neck squamous cell carcinoma; hRPE65, human retinal pigment epithelium 65 kDa; HSCT, hematopoietic stem cell transplantation; HSV, herpes simplex virus; NA, not available; MOH, Ministry of Health; NGFR, nerve growth factor receptor; PAD, peripheral artery disease; PFU, plaque forming unit; PMBCL, primary mediastinal large B-cell lymphoma; r/r, relapsed or refractory; SMA, spinal muscular atrophy; SMN, survival motor neuron; VEGF, vascular endothelial growth factor; VG, vector genome; VP, viral particle. All prices are in USD.*

**TABLE 3:** List of approved tissue-engineered products (TEPs).

No.	Trade name/proper name	Manufacturer	Indications	Approvedby/date	Dosage	Product form	Description	AT/AL	Price
1	Apligraf (Organogenesis Inc., 2019c, 2020)	Organogenesi, Inc. and Novartis AG (United States)	Chronic VLU, DFU	US FDA 2000 June	Circular disk with a diameter and thickness of 75 and 0.75 mm in size	Bag	Bi-layer bioengineered skin with inner layer of HDFn and outer layer of HEKn	AL	$1,500–2500 per treatment
2	Dermagraft (Organogenesis Inc., 2019d)	Organogenesis, Inc. (United States)	Full-thickness DFU > 6 weeks extended through the dermis without tendon, muscle, joint capsule, or bone exposure	US FDA 2001 September	2 × 3-inch sheets	Bag	Fibroblasts on a piece of bio-absorbable scaffold	AL	$1,700 per treatment
3	Aurix (Napodano, 2015; Nuo Therapeutics Inc., 2019)	Nuo Therapeutics, Inc. (United States)	All types of ulcers (DFU, pressure, VLU, etc.)	US FDA 2007 September	Depends on the size and condition of the wound	Gel	PRP hematogel	AL	$430 per treatment
4	Epicel Cultured epidermal autografts (Genzyme, 2019; Schlatter, 2019)	Vericel, Corp. (United States)	Deep dermal or full thickness burns	US FDA 2007 October	50 cm^2^ sheets with a thickness of 2–8 cell layers	Sheet	Cultured keratinocytes on murine 3T3 fibroblasts (each graft is attached to petrolatum gauze backing with titanium surgical clips)	AT	$6,000 to $10,000 per 1% TBSA
5	Gintuit ACKFBC (Food and Drug Administration, 2019o)	Organogenesis, Inc. (United States)	Surgically created vascular wound bed in the treatment of mucogingival conditions	US FDA 2012 March	Sheets with a diameter and thickness of 75 and 0.75 mm, respectively, which consist of ∼4 million cells	Sheet	Cultured neonatal keratinocytes and fibroblasts on bovine collagen	AL	NA
6	Omnigraft Dermal Regeneration Matrix (Food and Drug Administration, 2019p; Integra, 2019)	Integra LifeSciences, Corp. (United States)	DFU	US FDA 2016 January	Sheets with two sizes of 4 × 4 and 7 × 7 cm^2^	Sheet	Bi-layered bioengineered scaffold, including an inner layer of bovine collagen and chondroitin, and an outer layer consisting of thin silicone	Xn	$499.00 per Kit
7	MACI (Bloomberg Businessweek, 2018; Food and Drug Administration, 2019q)	Vericel, Corp. (United States)	Single or multiple symptomatic full-thickness cartilage defects of the knee with or without bone involvement in adults	US FDA 2016 December	3 × 5 cm^2^ sheets, consist of 500,000 cells per cm^2^	Sheet	Cultured chondrocytes on a porcine type I/III collagen membrane	AT	$40,000 per each scaffold
				EMA 2013 June					
8	Holoclar (EMC Inc., 2020; European Medicines Agency, 2020c)	Chiesi Farmaceutici S.p.A (Italy)	Severe limbal stem cell deficiency	EMA 2015 February	79,000–316,000 cells/cm^2^	Sheet	HCEpC containing stem cells	AT	∼ $102,977 per treatment per eye
9	Spherox (European Medicines Agency, 2020d; Startseite., 2020)	CO.DON AG (Germany)	Symptomatic articular cartilage defects of the femoral condyle and the patella of the knee with defect sizes up to 10 cm^2^ in adults	EMA 2017 July	10–70 spheroids/cm^2^	Tube	Tissue spheroids of human matrix-associated chondrocytes	AT	∼ $12224 per treatment
10	Holoderm (Kim, 2014; Food and Drug Administration, 2019i; Tego Science, 2019e)	Tego Science (South Korea)	Deep 2^*nd*^ and 3^*rd*^ degree burns	South Korea MFDS 2002 December	56 cm^2^/piece consisting of 1–4 billion keratinocytes	Sheet	Cultured keratinocyte sheet	AT	$697.76 per each cm^2^
11	Kaloderm (Jae-hyeon, 2018; Global Sources, 2019)	Tego Science (South Korea)	Deep 2^*nd*^ degree burns and DFU	South Korea MFDS 2005 March (for burns) 2010 January (for DFU)	Each sheet contains > 2 × 10^7^ cells backed by vaseline gauze. The amount is determined considering the size and condition of the wound.	Sheet	Cultured keratinocytes sheet	AL	$26.5 per 1 cm^2^ for products with 56 cm^2^ units
12	CardioCel Pure collagen scaffold (Taylor and Francis Group, 2015; Anteris Technologies Limited, 2019)	Admedus (Singapore)	ASD and VSD	Australia HAS 2014 November	Sheets with three sizes of 4 × 4, 5 × 8, and 14 × 7 cm^2^	Sheet	Tissue engineered bovine pericardium	Xn	$421.13 per sheet
13	JACE Epidermis-derived cell sheet (Drew, 2015; Pharmaceuticals and Medical Devices Agency, 2019)	J-TEC (Japan)	Scars, vitiligo, nevi (birthmarks), ulcers, skin-graft donor sites, severe burns: DDB + DB ≥ 30%	Japan PMDA 2007 October	A sheet consists of 1 × 10^4^ cells/cm^2^	Sheet	Keratinocytes sheet cultured on 3T3-J2 cells	AT	S$3,140 per 8 × 10 inch sheet
14	JACC AT Chondrocyte (Japan Tissue Engineering Co Ltd., 2020)	J-TEC (Japan)	Cartilage defect area >2–4 cm with no alternative therapy	Japan PMDA 2012 July	The quantity of mixture determined by the size of the cartilage defect: defect area x 0.3 ml (final cell density = 2 × 10^6^ cells/mL, 1.33% collagen)	Pre- field syringe	Cultured chondrocytes in atelocollagen gel	AT	$21,300 per knee
15	HeartSheet SDCS (Pharmaceuticals and Medical Devices Agency, 2019; Pricing of Approved Cell Therapy, 2019)	Terumo, Corp. (Japan)	Severe heart failure due to ischemic heart disease	Japan PMDA 2015 September	5 skeletal myoblast-derived cell sheets (containing 3 × 10^8^ cells)	Sheet	Skeletal myoblast sheet	AT	A kit: $56000 B kit: $15000 (Each treatment 1 A kit and 5 B kits)
16	Artificial transfected pigskin (Cheng et al., 2016)	Chongqing Zongshen Junhui Biotechnology (China)	Burns and other traumatic wounds	China CFDA 2007	NA	Sheet	Bama miniature pig fresh skin tissue transfected with CTLA4Ig gene	Xn	NA
17	Bilayer artificial skin (Cheng et al., 2016)	Shaanxi Eyre skin Biological Engineering (China)	Deep 2^*nd*^ degree burn wound, not more than 3^*rd*^ degree burn wound 20 cm^2^ (diameter <5 cm)	China CFDA 2007	NA	Sheet	A bilayer artificial skin with the epidermal layer composed of human epidermal cells and the dermal fibroblasts from human and bovine collagen	AL	NA
18	Amniosin (SinaCell Enterprise Knowledge Management, 2019)	SinaCell (Iran)	Corneal ulcer, full-thickness DFU > 6 weeks extended through the dermis without tendon, muscle, joint capsule, or bone exposure	Iran FDA 2017 March	One piece of 2 × 2 cm^2^ implanted	Sheet	Acellular human amniotic membrane-derived dressing	AL	NA
19	Cell-Amniosin (SinaCell Enterprise Knowledge Management, 2019)	SinaCell (Iran)	Full-thickness DFU > 6 weeks extended through the dermis without tendon, muscle, joint capsule, or bone exposure	Iran FDA 2017 March	One piece of 2 × 2, 3 × 3, 5 × 5, 5 × 10, or 10 × 10 cm^2^ implanted weekly until the ulcer is healed	Sheet	Cellular human amniotic membrane-derived dressing	AL	NA
20	Amniodisk (SinaCell Enterprise Knowledge Management, 2019)	SinaCell (Iran)	Corneal ulcer, conjunctival and epithelial damage	Iran FDA 2020 September	One piece of 15 mm circular shape sheet	Sheet	Dehydrated human amniotic membrane-derived ocular allograft	AL	NA

*ACKFBC, allogeneic cultured keratinocytes and fibroblasts in bovine collagen; AL, allogeneic; ASD, atrial septal defect; AT, autologous; CFDA, China Food and Drug Administration; CTLA4Ig, cytotoxic T-lymphocyte associated protein 4 immunoglobulin; DDB, deep dermal burn; DB, dermal burn; DFU, diabetic foot ulcer; EMA, European Medicines Agency; FDA, Food and Drug Administration; HAS, Heath Administration of Singapore; HDFn, human dermal fibroblasts (neonatal); HEKn, human epidermal keratinocytes (neonatal); MFDS, Ministry of Food and Drug Safety; NA, not available; PMDA, Pharmaceuticals and Medical Devices Agency; PRP, platelet-rich plasma; SDCS, skeletal myoblast-derived cell sheet; TBSA, total body surface area; VLU, venous leg ulcer; VSD, ventricular septal defect; Xn, xenogeneic. All prices are in USD.*

To achieving this end, published articles regarding the characteristics, safety and efficacy, and market size of approved ATMPs were reviewed using the search engines PubMed, Scopus, and Google Scholar. For some products which the related papers were not available, data on the relevant company website was used as reference. The type of documents used to obtain the data were original articles, review articles, HTML documents, and official websites of each product manufacturer. Search terms included MeSH (Medical Subject Headings) terms, “ATMP” “CTMP” “GTMP” “TEP” and also “product name” in addition to each terms of “efficacy” “safety” “adverse events” “price” and “market size.” The cut-off date for the data search was May 2020.

Collectively, this paper aims to provide a comprehensive insight for development of other cell therapy products for stakeholders, sponsors, manufacturing companies, regulatory agencies, and researchers interested in entering this research pathway.

## Classification of Advanced Therapy Medicinal Products

**FIGURE 1:**
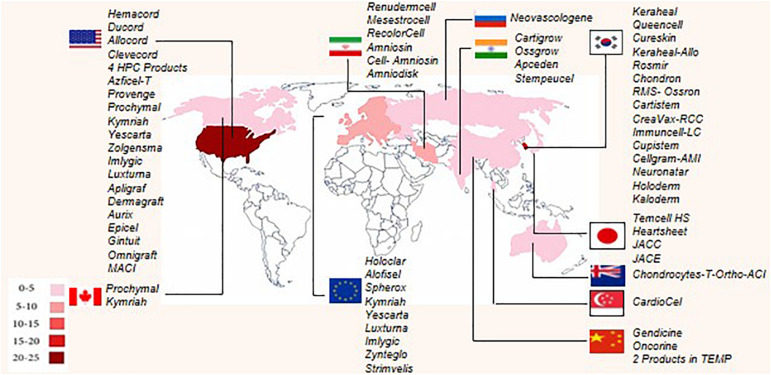
Number of authorized ATMPs worldwide, according to country. Each country has been determined by specific color, and based on the number of authorized ATMPs. United States, South Korea, and the European Union have the highest number of approved ATMPs.

**FIGURE 2:**
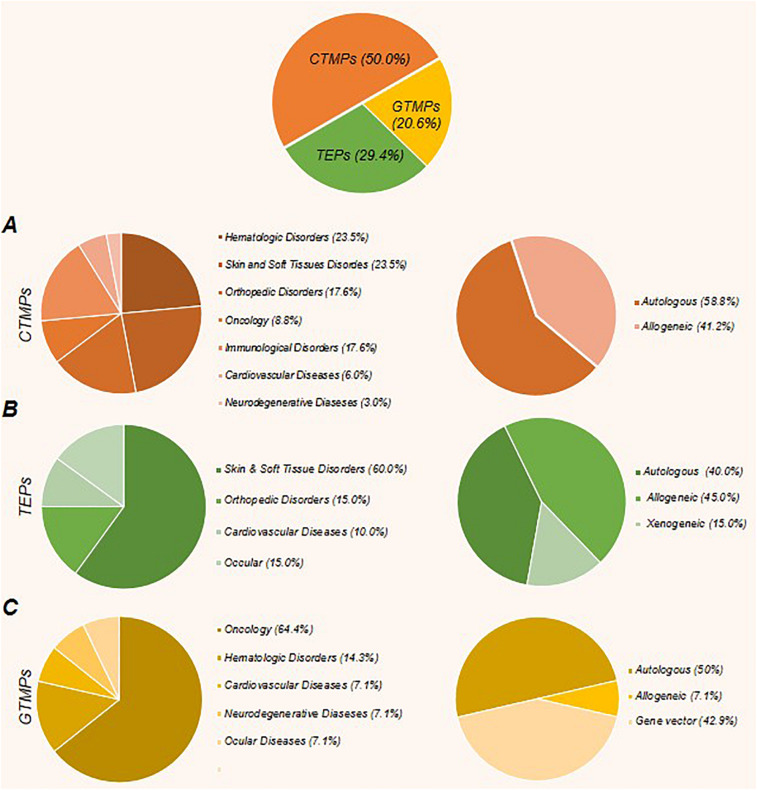
The percent of approved cell, gene, and tissue engineering products, along with their related indications and product type (autologous or allogeneic). **(A)** The CTMPs with 34 members constitute the largest class of ATMPs. Most of these products have an autologous source. Hematologic disorders, along with skin and soft tissue related disorders and orthopedic disorders constitute the main fields. **(B)** The TEPs, with 20 products, represent the next largest class of ATMPs. The most emphasized indications in this class are skin and soft tissue related disorders, orthopedic disorders, and cardiovascular diseases, respectively, and the percentage of allogeneic products are more than autologous products. **(C)** GTMPs with 10 products comprise the last class. The first common indications are oncology, hematologic disorders, and cardiovascular diseases, respectively. Similar to CTMPs, GTMP members mostly have autologous sources.

To the best of our knowledge, worldwide, there are 64 approved ATMPs by taking into consideration that Prochymal, a CTMP product, approved by both the FDA and Health Canada, and the GTMP product, Kymriah, is being approved by the FDA, EMA, and Health Canada. In addition, the FDA and EMA both approved another GTMP product, Yescarta. Obviously, the CTMP group, with 34 products, is the largest class. The TEP and GTMP groups, with 20 and 10 products, follow in order. Figure 1 shows that United States, by authorizing 23 ATMPs (11 CTMPs, 7 TEPs, and 5 GTMPs), is the pioneer country in this field followed by South Korea with 15 ATMPs (13 CTMPs and 2 TEPs). Figure 2 shows the indications of each of the ATMP categories, which emphasizes the importance of the indications related to hematologic along with skin and soft tissue disorders (Figure 2A), skin and soft tissue related disorders (Figure 2B), and oncology (Figure 2C) in the CTMPs, TEPs, and GTMPs, respectively. It can be concluded that most CTMPs and GTMPs have an autologous source (Figure 2A,C), while TEPs involve 45 percent of allogeneic and 40 percent of autologous products (Figure 2B).

## Safety and Efficacy of Advanced Therapy Medicinal Products (ATMPs)

### Cell Therapy Medicinal Products (CTMPs)

CTMPs can be divided into eight distinct groups with respect to the indication for which they have been developed: hematologic disorders, skin and soft tissue related issues, orthopedic diseases, oncology, and immunological, cardiovascular, neurological, and ocular related disorders.

**For hematologic disorders**, Hemacord (US FDA.2011), Ducord (US FDA.2012), Allocord (US FDA.2013), Clevecord (US FDA.2016), and four other HPC based products are approved for hematopoietic stem cell transplantations. Each product related efficacy has been compared against two studies: the COBLT study and another study with retrospective information from docket and public data (RxList, 2018; Food and Drug Administration, 2019b, c, d, e, f, g, h) with regards to neutrophil recovery at day 42, platelet recovery at day 100 (20,000/μL and 50,000/μL), and erythrocyte recovery at day 100. After receiving a TNC dose of ≥ 2.5 × 10^7^/kg HPC, from multiple cord blood banks, the estimated values were as follows: neutrophils (76%), platelets (20,000/μL [57%] and 50,000/μL [46%]), and erythrocyte (65%) recovery in the COBLT study. The docket and public data information show an estimated neutrophil recovery of 77% and platelet recovery (50,000/μL) of 45%. On the other hand, the three parameters for neutrophil recovery and platelet recovery (20,000/μL and 50,000/μL) parameters were 88, 87, and 79% for Allocord; 96, 92, and 83% for Clevecord; 95, 92, and 71% for Ducord; 79, 62, and 55% for HPCs Cord Blood from Clinimmune Labs; 88.2, 73.6, and 43% for HPCs Cord Blood from MD Anderson Cord Blood Bank; 91, 95, and, 95% for HPCs Cord Blood from LifeSouth; and 82, 66, and 50% for HPCs Cord Blood from Bloodworks, respectively. In addition, the reported data related to the Hemacord study were 83% (neutrophil) and 77% platelet (20,000/μL) recovery. The median time for neutrophil recovery, platelet recovery (20,000/μL and 50,000/μL), and erythrocyte recovery were 27, 90, 113, and 64 days in the COBLT study, respectively. The median time for neutrophil recovery and platelet recovery (50,000/μL) were 25 and 122 days according to docket and public data information. The median time of neutrophil recovery and platelet recovery (20,000/μL and 50,000/μL) were 21, 48, and 56 days for Allocord; 18, 41, and 43 days for Clevecord; 21, 46, and 61 days for Ducord; 25, 55, and 49 days for HPCs Cord Blood from Clinimmune labs; 19, 47, and 65 days for HPCs Cord Blood from MD Anderson Cord Blood Bank; 22, 44, and 70 days from HPCs Cord Blood from LifeSouth; and 21.5, 46, and 53 days for HPCs Cord Blood from Bloodworks, respectively. Also, 20 days for neutrophil recovery and 45 days for platelet recovery (20,000/μL) were reported for Hemacord. The most important adverse events related to the safety of these products included hypersensitivity reactions, infusion reactions, graft-versus-host disease, engraftment syndrome and graft failure, malignancies of donor origin, transmission of serious infections, and transmission of rare genetic diseases.

**For skin and soft tissue related disorders**, CureSkin (South Korea MFDS.2010), Queencell (South Korea MFDS.2010), Azficel-T (US FDA.2011), Rosmir (South Korea MFDS.2017), and RenudermCell (Iran FDA.2018) are approved to treat acne scars and facial wrinkles, KeraHeal (South Korea MFDS.2006) and KeraHeal-allo (South Korea MFDS.2015) are approved for skin burns and finally RecolorCell (Iran FDA.2019) is indicated for different types of Vitiligo. The effectiveness of Azficel-T was demonstrated in two studies consist of 421 total patients at 3 and 6 months follow up. The self- and physician-reported assessments indicated 57 and 33% improvements in the first study, and 45 and 19% in the second study for patients who received Azficel-T. A two-point improvement in NLF wrinkles was reported after 6 months. The most common adverse events were injection-site redness, bruising, swelling, pain, hemorrhage, edema, nodules, papules, irritation, dermatitis, and pruritus (S-Biomedics Ltd., 2019a). Qualitative data regarding the efficacy of CureSkin shows its superior effect in the healing process of depressed acne scars (S-Biomedics Ltd., 2012). Moreover, according to the product’s brochure, the most common reported adverse events after repeated administration of CureSkin was erythema at the injection site. Common adverse events for Rosmir included eye irritation and allergic rhinitis (Yim et al., 2011; Tego Science, 2019a). Regarding KeraHeal efficacy, the take rate of 1:4–6 meshed autografts along with KeraHeal in 29 patients with burn injuries was estimated to be 96 and 100% at 2 and 4 weeks after treatment, respectively. A reduction in Vancouver burn scar scale at 8, 12, and 24 weeks following the treatment was observed (Yoon et al., 2017). The primary outcome used to show the KeraHeal-allo efficacy was the period of re-epithelialization, which occurred 2.5 or 2.8 days faster in the treated sites in comparison with the control. No associated adverse events were reported with these two products (Park et al., 2017).

**For orthopedic disorders**, Cartistem (South Korea MFDS.2012) and MesestroCell (Iran FDA.2018) are approved for knee osteoarthritis; Chondron (South Korea MFDS.2001), Cartigrow (India DCGI.2017), and Chondrocytes-T-Ortho-ACI (Australia TGA.2017) are approved for defective knee cartilage. Ossron (South Korea MFDS.2009) and Ossgrow (India DCGI.2017) are approved for repair of bone defects. The safety and efficacy of Cartistem was assessed in a phase I/II clinical trial with 7 years long−term follow−up in seven patients with osteoarthritis of the knee joint with Kellgren−Lawrence (K−L) grade 3 and painful full−thickness cartilage defects. Results have shown repaired tissue at 12-weeks post-transplantation, while arthroscopic examination and biopsy at 1 year showed stable regenerated cartilage. Furthermore, the 100-mm VAS score for pain and the IKDC subjective score were improved at 24 weeks post-transplantation and maintained for up to 7 years. A high glycosaminoglycan content in the regenerated cartilage at 3 years was determined through the mean relative change in R1 (ΔR1) index. Mild to moderate adverse events included arthralgia, back pain, and bladder distension. No particular adverse reactions were noted over 7 years of clinical follow-up (Choi et al., 2010). Following the Chondron transplantation in 98 patients with articular cartilage injury of the knee joint and 13∼25-month follow-up, assessments showed a notable improvement in the Knee Society Scoring system (tKSS)-A (pain) and tKSS-B (function) scores. A total of 2.04% of the patients experienced adverse events related to GACI due to ‘catching symptom’ (Bauer et al., 2012). The safety, tolerability, and efficacy of Chondrocytes-T-Ortho-ACI are reported from clinical trials of 1077 reported cases that showed significant improvements in the KOOS subscales and in the 6MWT during 36 months of post-surgery follow up in comparison with the pre-operative group. MRI analysis also showed significant post-operative progression; however, this observation was not sustained until the study end point. The most common adverse events encountered during these clinical trials included engraft failure, cartilage hypertrophy, incomplete drug effect, and graft delamination (Kim et al., 2009). Safety and efficacy of Ossron and Ossgrow have been assessed in a clinical trial of 64 patients with long-bone shaft fractures during 2 months. The average callus formation score was significantly higher in the experimental group at 1 and 2 months of follow up, while the osteoblast injection response was not statistically different between younger and older patients. No adverse effects were observed in association with the osteoblast injection (Kim et al., 2007).

**In oncology,** CreaVax-RCC (South Korea MFDS.2007) is approved for metastatic renal cell carcinoma, Immuncell-LC (South Korea MFDS.2007) is approved for post-surgical recurrence of hepatocellular carcinoma, Sipuleucel-T (Provenge) (US FDA.2010/EMA.2010) is approved for mCRPC, and finally, Apceden (India DCGI.2017) is approved for Prostate, ovarian, colorectal, and NSCLC. The safety and efficacy of CreaVax-RCC was assessed in nine patients suffering from metastatic renal cell carcinoma with a median follow-up of 17.5 months. This treatment had the ability to prompt an immune response against the tumor. Out of nine patients, one experienced a PR and a decrease in the size of lung metastases; five had stable disease; and three had evidence of progressive disease after one cycle of immunotherapy. The results of the DTH skin test with KLH or TL-pulsed DCs determined that three patients with no initial DTH reactivity and three patients with a positive initial DTH response to KLH- or TL-pulsed DC had positive reactions to both after immunotherapy and experienced raised skin reactions after the vaccination. In addition, there was an elevation in the number of tumor specific interferon gamma (IFN-γ)-producing cells after one cycle of the vaccination. No severe adverse effects have been reported (Lee et al., 2015). In the Immuncell-LC phase III clinical trial, 230 patients with HCC were assigned randomly to receive immunotherapy 16 times during 60 weeks or no adjuvant therapy (controls). RFS and RFS rates were considered to be the primary outcomes. Overall survival (OS), cancer-specific survival, the OS rate, and cancer-specific survival rate criteria accounted for secondary outcomes. The RFS time for the immunotherapy group was 44 months in comparison with 30 months in the control group. The RFS rate for both groups declined during 12, 24, 36, and 48 months post-treatment; however, the amount of statistically significant rates per month were higher in the immunotherapy group. Both the OS rate and cancer-specific survival rate decreased in the immunotherapy and control groups during 12, 24, 36, and 48 months. Again, the amounts of the statistical rates per month were higher in the treatment group. Serious adverse events were reported in the immunotherapy group and included hemorrhage from esophageal varices, hepatic vein stenosis, herpes zoster, laceration, meniscus lesion, humerus fracture, foot fracture, bladder neoplasm, and high frequency ablation (Kantoff et al., 2010). In a phase III clinical trial, 341 patients with mCRPC received Stempeucel-T (Provenge). There was a relative decline of 22% in the risk of death observed in the sipuleucel-T group compared with the placebo group. This reduction resulted in a 4.1-month improvement in median patient survival. The most common adverse events were chills, fever, and headache. These adverse events were more frequent in the sipuleucel-T group than in the placebo group (Bapsy et al., 2014). Safety and efficacy of Apceden was assessed in a multicenter phase II clinical trial in India that enrolled 51 patients with refractory solid cancers. A significant improvement regarding QOL and overall median survival in patients with objective response was observed. The TTP analysis showed a notable delay in the onset of disease development. There was an increase in the mean CD4:CD8 ratio in the immune response evaluation, along with an ORR of 28.9 and 42.1% by RECIST and irRC, respectively. One adverse event, an episode of rigors together with mild fever during a single infusion was reported (Prasad et al., 2011).

**Immunological disorders** have four approved products. Prochymal (Health Canada.2012, US FDA.2015) together with Temcell HS (Japan PMDA.2015) are produced to treat GvHD. Cupistem (South Korea MFDS.2012) and Alofisel (EMA.2018) are two other products in this group. First one is indicated for reducing the inflammation in Crohn’s Fistula and the latter is indicated for treatment of complex perianal fistulas in adult patients with non-active/mildly active luminal Crohn’s disease. In one trial, Prochymal was used to treat refractory grades III and IV acute GvHD in 12 children. The results indicated that allogeneic HSCT was well-tolerated. The survival rate was 42% after a median follow up of 611 days. The OS for patients who achieved CR was estimated to be 68% at 2 years (Muroi et al., 2016). Temcell HS was evaluated in a phase I/II study of 25 patients with steroid-refractory grade III or IV acute GvHD. A statistically significant consistent CR for grade III or IV steroid-refractory acute GvHD was shown from 4 to 52 weeks of follow up. At 52 weeks, 48% of the patients who achieved CR were still alive. In addition, the survival of patients who had an OR, which is the sum of the CR and PR, was substantially higher than those with no OR. Responses in children were better than adults. The most common adverse events were leukocytopenia, thrombocytopenia, anemia, sepsis, hypertension, microangiopathy, liver dysfunction, and chronic GvHD (European Medicines Agency, 2020a).

The assessment efficacy and safety of Alofisel compared to placebo was considered in a pivotal Phase III clinical trial. In this study 212 perianal fistulising CD patients (107 receiving Alofisel Cx601 and 105 receiving placebo) was screened over 24, 52, and 104 weeks. Full analysis of the efficacy data showed combined remission of perianal fistulising CD and absence of collections > 2 cm of the treated fistula confirmed by MRI images, at week 24. The combined remission in the active group was 49.5% (53/107) and in the placebo group were 34.5% (36/105). Presented data from week 52 showed statistically significant effects in favor of Alofisel treatment, and finally in patients who entered the 104 weeks follow-up (25 Alofisel, 15 placebo), the rate of clinical remission was 56 and 40% in the active and placebo group, respectively (Cho et al., 2015). In a phase II clinical trial fistula healing was evaluated 24 months after the administration of Cupistem in 43 patients. Cupistem seemed to be efficient considering the results of the mPP analysis 24 months after transplantation where 80.8% of the patients showed complete fistula healing. In order to assess the sustainability of the initial response, complete closure in 83.3% of the 24 patients who had evidence of complete closure at the 8^*th*^ week after the injection still had complete closure at year 2. The most common adverse reactions were abdominal pain, eczema and exacerbation of crohn’s disease, anal inflammation, diarrhea, and fever. None of the observed adverse events were considered to be related to the product (Kim et al., 2018).

**Cardiovascular diseases** are the next category in the field of CTMPs. Cellgram-AMI (South Korea MFDS.2011) for AMI and Stempeucel (India DCGI.2016) for CLI due to thromboangiitis obliterans (Buerger’s disease) are in this group. In a clinical study, for evaluating the safety and efficacy of Cellgram-AMI, 26 patients with successful PCI for acute ST-segment elevation anterior wall myocardial infarction were assigned to either a control group (*n* = 12) or Cellgram group (*n* = 14) and were follow-up for 4-month. Patients who received Cellgram-AMI had improved Left ventricular function as shown by a substantial progress in overall LVEF, measured by SPECT and echocardiography 3 months after the BM-MSC injection and 4 months after PCI. This improvement continued to the 12^*th*^ month follow up as assessed by echocardiography. However, the baseline and 4-month LVEDV and LVESV values did not significantly change. No adverse events, in-stent restenosis, or proarrhythmic effects were noted in both groups during the 4 and 12-month follow up periods (Gupta et al., 2017). A phase II study in India assessed the safety and efficacy of Stempeucel. This study placed 90 patients with CLI due to Buerger’s disease into two dose groups. The rest pain and ulcer size per month were the primary outcomes. Both decreased in comparison with the SOC group. The secondary outcomes of ABPI, amount of total walking distance, and QOL activity score of units per month increased for both doses. Skin ulcer and gangrene in the 1 × 10^6^ and 2 × 10^6^cells/kg groups, respectively, were the most frequently reported TEAEs. They were considered either remotely related or unrelated to Stempeucel (Oh et al., 2015).

The only related **neurological disorder** was ALS for which the product Neuronata-R (South Korea MFDS.2014) has been approved. In a phase I clinical trial to evaluate the safety of of Neuronata-R, seven patients with definite or probable ALS received two intrathecal injections of Neuronata-R and were follow-up for 12 months. The ALSFRS score, AALS score, and FVC were used to assess treatment efficacy. It was shown that none of the mentioned parameters declined rapidly and that the decrease in ALSFRS-R score during the 6 months follow up was more gradual than the observed decrease in the lead-in period, while these scores remained persistent for 6 months after the first injection of MSCs. None of the patients experienced serious adverse events during the 12-month follow-up period (Zhang et al., 2018).

### Gene Therapy Medicinal Products (GTMPs)

The GTMPs have been developed for oncology, hematologic, cardiovascular, neurodegenerative, and ocular diseases.

**In oncology related indications**, Gendicine (CFDA.2003) is the first approved gene therapy product to treat head and neck squamous cell carcinoma, Imlygic (US FDA/EMA.2015) is approved for melanoma treatment, and Kymriah (US FDA.2017-EMA/Health Canada.2018) and Yescarta (US FDA.2017-EMA.2018) are two products used for hematologic malignancies. To evaluate the safety and efficacy of Gendicine, there are over 30 clinical study related publications. This product was assessed in a large number of clinical studies that included more than 30,000 patients. The results showed remarkable safety records along with improvements in efficacy, including tumor shrinkage and an enhanced QOL. The average response rate for CR and PR reached 90%. After the 5-year follow up, a large group of patients were still alive. The most common adverse events include fever, arthralgia, and myalgia (Andtbacka et al., 2015). In a phase III clinical trial, an intralesional injection of Imlygic was compared with subcutaneous administration of GM-CSF in patients with advanced melanoma. The mean treatment duration was 23 in the Imlygic group and 10 weeks in the GM-CSF group. The median OS was determined to be 23.3 in the patients treated with Imlygic compared with 18.9 months in patients who received GM-CSF. The DRR and ORR were considerably higher in the Imlygic arm. Additional efficacy criteria included the median TTF and median time to response which were 8.2 and 4.1 months in Imlygic treated group versus 2.9 and 3.7 months in the GM-CSF arm. The most common reported adverse events were fatigue, chills, and pyrexia (Maude et al., 2018). In a phase II clinical trial, 75 pediatric and young adults with relapsed or refractory B-ALL received Kymriah. The ORR was 81% at 3 months of follow-up. EFS and OS rates were calculated 73 and 90% at 6 months, and 50 and 76% at 12 months, respectively. The median duration of remission and EFS were not reached, while the rate for RFS was 80 and 59% at 6 and 12 months, respectively, among patients who responded to treatment. The most common non-hematological adverse reaction in 77% of patients was cytokine release syndrome. Also, neurologic events occurred in 40% of patients (Schuster et al., 2019). In another phase II clinical trial, to evaluate the efficacy of Kymriah therapy, 93 patients with relapsed or refractory DLBCL were infused with Kymriah and the median follow up time was 14 months. The best OR was 52%, 40% of the patients had complete responses and 12% had partial responses in a median of 2 months. After 12 months of the initial response, the rate of RFS was estimated to be 65% (79% among patients with a complete response). Also, cytokine release syndrome (58%), anemia (48%), and pyrexia (35%) were the most common adverse events of any grade (Neelapu et al., 2017). In a phase II clinical trial, Yescarta was administrated to 101 patients with DLBCL and PMBCL. The ORR and the complete response rate were 82 and 54%, respectively. The OS rates at 6, 12, and 18 months were 78, 59, and 52%. CAR T cell levels in the blood peaked within 14 days and were detectable in most patients at 180 days after the Yescarta infusion. The most serious adverse reactions included cytokine release syndrome and neurologic events (Cicalese et al., 2016).

**For hematologic disorders,** Strimvelis (EMA.2016) is developed to treat adenosine deaminase deficiency derived severe combined immunodeficiency (ADA-SCID) and Zynteglo (EMA.2019) is approved to treat Patients up to 12 years old with beta thalassemia who require regular blood transfusions.

In a phase II clinical trial, 18 patients with ADA-SCID received Strimvelis. The OS in a median follow-up of 6.9 years was 100% and there were increased numbers of CD3 +, CD4 +, CD8 + T cells, and CD16 + CD56 + NK cells as an outcome of immune reconstitution. A slower increase in CD19 + B-cells was reported. The TREC and lymphocyte ADA enzyme activity both increased in peripheral blood lymphocytes after treatment. Venous red blood cell deoxyadenosine nucleotide levels were <100 nmol/ml. The most frequent adverse events were respiratory and gastrointestinal tract infections (Karponi and Zogas, 2019). Five completed and ongoing clinical trials are existed regarding Zynteglo, HGB-205, HGB-204, HGB-207, HGB-212, and LTF-303. During HGB-204 and HGB-205 clinical trials, it was revealed that 11 of 18 and all of 4 enrolled patients with transfusion-dependent beta (β)-thalassemia (TDT) in each study met the primary endpoint which was the elimination of RBC transfusion requirement, respectively. HGB-207, HGB-212 clinical trials and a long-term follow-up study named LTF-303 constitute the ongoing clinical trials. So far, 17 of 20 and 6 of 11 enrolled patients in HGB-207, HGB-212 clinical trials have shown transfusion independency, respectively (European Medicines Agency, 2020e). Thrombocytopenia constituted the only serious adverse reaction related to Zynteglo. Moreover, there were common adverse reactions attributed to Zynteglo containing leukopenia, neutropenia, hot flush, dyspnea, pain in extremity, non-cardiac chest pain, and one very common adverse reaction as abdominal pain (ClinicalTrials, 2020).

Some of the common side effects reported in patients receiving Zynteglo^TM^ during the clinical trials were a low count of thrombocytes, numbness in hands and feet, pain in the bone, nausea, headache, and low blood calcium levels (Deev et al., 2018).

A phase I/IIa clinical trial for Neovasculgen (Russian MOH.2011), as the single product in the **cardiovascular diseases class**, verified the safety of this product. In phase IIb/III trials that enrolled 100 patients (75 in the treatment and 25 in the control group), PWD was estimated to have increased significantly by 110% at 6 months in the treatment group. Moreover, PWD increased in the Neovasculgen treated group by 167% at 1 year and 191% at 2 years after treatment. There were no reported adverse events (Deev et al., 2015a, 2017; NOVARTIS, 2019a). Finally, the safety and efficacy of Zolgensma (US FDA.2019) one of the recent gene therapy products for **neurodegenerative related diseases** is being evaluated in an ongoing phase III STR1VE trial that enrolled 21 SMA pediatric patients with biallelic mutations in the survival motor neuron (SMN1) gene. As of the March 2019 data cutoff, remarkable survival rates, improved rapid motor function, and the capability to sit without support were among the most momentous results related to the efficacy of this product. The most common adverse events were elevated aminotransferases and vomiting (Russell et al., 2017). Luxturna (US FDA.2017/EMA.2018), the only gene product related to **ocular diseases** was assessed in a phase III clinical trial of 31 patients with RPE65-mediated inherited retinal dystrophy. The mean bilateral MLMT score was 1.8 light levels in the intervention group and 0.2 in the control group. Mean FST improved by more than two log units by day 30 in the intervention group, whereas the control group had no meaningful change. However, BCVA showed a numerical improvement among both groups. The most common adverse events included increased intraocular pressure, cataracts, retinal tears, and eye inflammation (Edmonds, 2009).

### Tissue-Engineered Products (TEPs)

Skin and soft tissue related disorders, orthopedic, cardiovascular and ocular disorders are four indications for authorized TEPs.

**For skin and soft tissue disorders**, Apligraf (US FDA.2000), Dermagraft (US FDA.2001), Aurix (US FDA.2007), Omnigraft (US FDA.2016), Amniocin (Iran FDA.2017), and Cell-Amniosin (Iran FDA.2017) are developed to treat Chronic VLU and/or DFU. Holoderm (South Korea MFDS.2002), Epicel (US FDA.2007), and JACE (Japan PMDA.2007) are approved for skin burns and finally, Kaloderm (South Korea MFDS.2005, for burns/2010, DFU) is approved for both DFU and deep 2^*nd*^ degree burns. The safety and efficacy Apligraf compared to standard therapy was assessed in 106 patients with neuropathic DFUs during 12 weeks. Kaplan–Meier curves indicated that the Apligraf treated group had a significantly faster complete wound closure in comparison with the standard treatment; after 12 weeks, 51.5% of patients who received Apligraf had achieved complete wound closure compared with 26.3% in the control group. The reported related adverse events consist of suspected wound local infection, cellulitis, and exudate (Marston et al., 2003). A clinical study of 314 patients with chronic DFUs evaluated the safety and efficacy of Dermagraft. The results showed a trend toward a shorter time for complete wound healing using Dermagraft. In addition, 30.0% of Dermagraft patients achieved complete wound closure compared with 18.3% of control patients after 12 weeks. No specific related adverse events were reported and the incidence of ulcer infection, cellulitis, or osteomyelitis was significantly lower in the Dermagraft treated patients versus the control patients (Driver et al., 2006). A clinical study of Aurix for 72 patients who suffered from non-healing DFU showed that 91% of long-term non-healing wounds responded to treatment with a 64% reduction in volume during 15 days or less. They researchers observed that 81.3% of Aurix treated wounds, which were less than 7 cm^2^, healed completely within 6.2 weeks in comparison with saline gel. In addition, the Kaplan–Meier time-to-healing was significantly better in the Aurix group. No product-related serious adverse events were reported (Driver et al., 2015). The results of the study that evaluated the safety and efficacy of Omnigraft in comparison to standard wound care in 307 patients with neuropathic DFU demonstrated improved life quality, approximately 5 weeks faster wound closure, and 19% increased healing rate in patients treated with Omnigraft (Tego Science, 2019b). Allergic reaction was the most concern about the safety of Omnigraft. We were unable to locate any data that pertained to the efficacy of Holoderm; however, according to the product brochure, abnormal cellular responses such as dyskeratosis or parakeratosis may occur following the use of Holoderm-derived epidermis (Food and Drug Administration, 2019i). The survival rate of Epicel was assessed in three studies. In the first study, the OS rate was 86.6% for overall patients and 89.3% for pediatric patients at 3 months after the initial implantation. In the second study, an OS rate of 88.3% in pediatric patients compared with 81.3% in the total population was reported. In the third study, the treatment group had a 90% survival rate compared to 37.5% for the control group. The most common adverse reactions were infections, graft shear, blisters, drainage, sepsis, graft detachment, and renal failure (Matsumura et al., 2016). The CEA JACE safety and efficacy was evaluated through a 6-year multicenter clinical trial for treatment of burns that covered more than 30% of the TBSA. The mean CEA take rate at 4 weeks post-engraftment was 66%, while the use of combined treatments such as artificial dermis or a wide split-thickness auto or a patch graft significantly elevated this rate. The most common adverse events were skin ulcers or auto graft detachment; however, death and sepsis, which were reported as serious adverse events during later periods, did not appear to be related to CEA (Tego Science, 2019c). Treatment of DFU with Kaloderm has shown that 12 weeks after treatment, all patients in the keratinocyte-treated group and 69% of patients in the control group experienced complete wound healing. No adverse events were reported in relation with the wound dressings. In terms of skin burns, the product packaging insert for Kaloderm stated that, no adverse reaction has been reported other than a possible occasional infection at the site, dermatitis, exudate formation, weak edema, hypersensitivity, and pain. In addition, Kaloderm can promote the re-epithelialization of deep abdominal cavity burns (McGuire et al., 2011; You et al., 2012).

Gintuit (US FDA.2012) is approved for surgically created **vascular wound** in the treatment of mucogingival conditions, the results of a clinical trial with 96 patients during 6 months follow up showed that the LCC mediated ≥ 2 mm regenerated keratinized gingiva in 81 of 85 patients and ≥ 1 mm in all patients, while the color and texture was similar to the adjacent tissue. The most common adverse events included sinusitis, nasopharyngitis, respiratory tract infections, aphthous stomatitis, and the local effects of oral surgery (Tohyama et al., 2009).

**In orthopedics disorders**, JACC (Japan PMDA.2012), MACI (US FDA.2016/EMA.2013) and Spherox (EMA.2017), are used to treat cartilage defects. JACC was studied through a multi-center clinical trial for transplanting autologous cultured chondrocytes in 27 patients with cartilage lesions who were evaluated at 3, 6, 12, and 24 months after the implantation surgery. Elimination of locked knee together with decreased pain were observed following the transplantation. Also, substantial progression of the original knee-function scale and the clinical scores based on the Lysholm Knee Scoring Scale, and observation of the natural appearance in 92% of patients as indicated by arthroscopic assessment showed restorative promotion of articular cartilage in the knees (Saris et al., 2014). There were few adverse events, except for two cases of graft detachment. The efficiency of a MACI implant has been assessed and its superiority versus microfracture treatment was evaluated in the SUMMIT clinical trial of 144 patients. The results at week 104 revealed significant improvement with MACI in the three KOOS subscales of pain, SRA, and ADL when compared with the microfracture group. Serious adverse reactions reported for MACI were arthralgia, cartilage injury, meniscus injury, treatment failure, and osteoarthritis (Armoiry et al., 2018). In a phase III clinical trial, Spherox was compared with the microfracture treatment in 102 patients. The mean overall KOOS in patients treated with Spherox increased from 56.6 ± 15.4 at baseline to 78.7 ± 18.6 after 12 months, with a further increase to 81.5 ± 17.3 after 24 months. However, the MOCART scores did not change significantly among the two groups (Becher et al., 2017). In a phase II clinical trial, 73 patients with cartilage defects received transplants of three different doses (low, medium, and high) and were subsequently followed until 36 months. Severe adverse events included meniscus lesion with the low dose; syncope and joint effusion with the medium dose; and arthralgia, joint effusion, and chondropathy with the high dose (Nordmeyer et al., 2018).

**For cardiovascular diseases**, CardioCel (Singapore HAS.2014) is approved to treat ASD and VSD and Heartsheet (Japan PMDA.2015) is developed for severe heart failure due to ischemic heart disease. In a study, CardioCel patches were applied on 40 patients for 2 years. While the probability of stopping the development of the combined end point that consisted of death, additional surgery, and a moderate degree of aortic valve dysfunction after AVR was 92 ± 5% at 12 months, this probability reduced to 28 ± 9% at 36 months after surgery. In this study, 23% of patients experienced an event during follow up, which included death and additional surgery due to stenosis, aortic valve insufficiency, and aortic valve endocarditis (Imamura et al., 2016). Heartsheet was evaluated in a phase II multi-center clinical trial of autologous skeletal myoblast sheet transplantation in seven patients with advanced heart failure compared with a control group receiving CRT in a 1 year follow upLVRR and heart failure symptoms improved in the treatment group and a lower rate of cardiac death during 800 days of follow up was observed. Common adverse events experienced by all of the patients during the study included arrhythmia, wound complications, hypokalemia, and post-operative fever (Rama et al., 2010). **For ocular disorder**, Amniodisk (Iran FDA.2020) is approved for Corneal ulcer, conjunctival and epithelial damage and Holoclar (EMA.2015) is approved for severe limbal stem cell deficiency. In a retrospective case series study, 106 patients with corneal damage received Holoclar. As the human limbal stem cells are recognized through p63 transcription factor expression, the clinical outcome assessment was conducted according to the percentage of p63-bright holoclone-forming stem cells in culture. If this percentage was greater than 3%, then the transplantation was considered successful. The success, partial success, and failure rates in the transplantation process were 76.6, 13.1, and 10.3% of the treated eyes, respectively (European Medicines Agency, 2015). The most common adverse reactions were blepharitis and corneal epithelium defects (Market Research, 2020).

## Market Size of Advanced Therapy Medicinal Products (ATMPs)

Price and market size are two main issues that should be emphasized for guarantee of product survival in the market. We have attempted to provide information of the market size of most products introduced in this paper by preferably accessing the appropriate company‘s available data on the Internet. All product prices are presented in USD to be comparable. The information regarding the market size of each mentioned product was collected by using company’s IR book, website, and market research websites. The disease markets were considered based on the related CAGRs, following with the data respecting each product.

Thus far, the area of diseases that is targeted by ATMPs are divided into hematological disorders, skin and soft tissue disorders, orthopedic disorders, immunological disorders, cardiovascular diseases, neurodegenerative diseases, ocular diseases, and cancers. The market size of each mentioned diseases can be classified based on the industrial analysis and CAGR. According to Mordorintelligence, the largest predicted CAGR from 2020 till 2025 belongs to dermatology (Cancer Therapy Market, 2020) therapeutics market (8.95%) and cancers (Autoimmune Disease Diagnostics Market, 2020), autoimmune disorders (Orthopedic Devices Market, 2020), orthopedic disorders (Cardiovascular Devices Market, 2020), cardiovascular diseases (CellTech, 2019) and the hematology (Hematology Market, 2020) field come in the next places with the CAGRs of 8.37, 8.3, 7.2, 6.2, and 5%, respectively. Also, predicted CAGRs for markets of soft tissue (Soft Tissue Repair Market, 2020) repair, neurodegenerative diseases (Neurodegenerative Disease Market, 2020), and ophthalmic disorders (Ophthalmic Drugs Market, 2020) from 2019 till 2024 are 5.8, 5.5, and 4.6%. Therefore, beginning with dermatology therapeutics, Fibrocell Science has announced that the sale of Azficel-T increased approximately $0.3 million, or 63.4%, for the year ending December 31, 2016 compared to 2015 (Fibrocell Science Inc., 2020) Apligraf and Dermagraft have experienced strong sales growth, with more than one million units of each one, shipped for patient use until April 2016 (Organogenesis Inc., 2019a) along with approximately one million patient units for Apligraf until September 2017 (Organogenesis Inc., 2019b). Nuo Therapeutics estimated a turn in the Aurix sales from $0.495 million in 2014, to peak sales over $50 million (Napodano, 2015). Based on Vericel investor reports, Epicel has been administered to approximately 100 patients in the U.S. annually, and it could achieve $23.1 million net revenue by December 31, 2018 (Vericel Corporation, 2019). According to the company’s IR book, the operating margin of Tego Science Company for Kaloderm and Holoderm products was $10.9 million in 2006, which reached $23.4 million in 2013. Sales of Kaloderm were lower in 2006; however, by 2013, sales of Kaloderm were significantly higher than Holoderm (Tego Science, 2019d). As stated by Biosolution Company’s IR book, 15% of the market in 2008 in South Korea belonged to KeraHeal in comparison with competitive products. This amount rose to more than 70% in 2012. Regarding the market size, the number of patients in 2017 was approximately 410 with the sales percent of nearly $9.5 million. The number of patients is expected to reach approximately 450 in 2021 with an estimated sales of $42.6 million (Bio Solution Co Ltd., 2019). In 2017, the first year that KeraHeal-Allo was released, it occupied 20% of the market. It was estimated that this product would experience rapid growth in sales during 2019. Regarding market size, in 2017 there were approximately 203,000 persons with a sale of $7.5 million. The numbers of patients are expected to reach 217,000 persons in 2021 with an estimated sales of $56 million (Bio Solution Co Ltd., 2019). The J-TEC company in 2009 has estimated that the market size of JACE would be approximately 100 to 300 million US dollars only in Japan (Joo-sung, 2016). In the cancer area, the Green Cross Cell company IR book estimated that the market potential for Immuncell-LC for only the US will be about $6 billion, with the targeted market consisting of liver cancer, brain tumors, and pancreatic cancer (Green Cross Cell, 2019). Also, several approved GTMP therapeutics, Kymriah, Yescarta, Imlygic, Zalmoxis, Gendicine, and Oncorine are indicated for oncology related diseases. Kymriah grew strongly in Europe and US in the first quarter of 2020, with a net sale of $93 million (Globenewswire, 2020). Also, Yescarta’s sale during the first quarter of 2019 was $96 million compared to $40 million for the same period in 2018 (Business Wire, 2020). Amgen, the manufacturer of Imlygic, has not disclosed Imlygic sales in its quarterly results presentations. However, EvaluatePharma reported consensus analyst forecasts of $45 million and $250 million for worldwide Imlygic sales in 2016 and 2022, respectively (Imlygic, 2017). Financial reports in 2012 revealed that over 6000 patients diagnosed with various types of solid cancers in China received Gendicin, which had a CAGR from 2004 to 2011 of 68.3% (Kudrin, 2018). Sales of Gendicin were $3.6 million in 2007 and $16 million in 2008. However, the sales of Oncorine were unsatisfactory in China (<$1.2) due to its high price in 2009 (Kudrin, 2018). Products indicted for immunological disorders show that Alofisel is forecasted by Evaluate Pharma that the worldwide consensus sales will reach $529 million in 2024 (Mesoblast Limited, 2020). In terms of the Cupistem market in South Korea, the number of CD patients increased from approximately 15,000 in 2010 to almost 20,000 in 2014, while an increased prevalence has also been observed (Green Cross Cell, 2019). Sales of Temcell HS were $14.3 million in FY 2017, which showed a 124.3% increase compared to previous fiscal years (HIYAKU, 2019). For orthopedic disorders, in the beginning of 2019, Cartistem sold $11.1 million and the revenues for its sale accounted for 34.4% of the total sales in the first 6 months of 2019 (Korea Biomedical, 2020). Chondron sales for cartilage cell therapy in 2012 were approximately $13.8 million and increased to approximately $17.8 million in 2014 (Mksvc, 2019). In addition, Cartigrow has been used in 140,000 knee replacement cases annually (Das, 2018). According to Orthocel, Chondrocytes-T-Ortho-ACI is currently marketed in Australia, New Zealand, Hong Kong, Singapore, the United States, Europe, and China. Total revenues for its sale reached $739,100 during the 2018 fiscal year (FY). Moreover, the revenue forecast for Chondrocytes-T-Ortho-ACI until the FY 2028 is an estimated $340,203,044 (Hice, 2018). According to a 2018 report published in the standard business website, Ossgrow is used in 108,000 cases of total hip replacement in India each year (Das, 2018). TMACI, Spherox, and JACC are other approved therapeutics for orthopedic disorders. Vericel Company announced that MACI generated net revenues of approximately $67.7 million in the year ending December 31, 2018 (Vericel Corporation, 2019). CO.DON AG, launched Spherox for distribution in European countries in September 2017, starting from Germany. In Germany, the market volume addressed by CO.DON AG, approximated 20,000 annual treatments. With a population of 82.2 million, this meant that in Germany about 0.025% of the population could receive treatment with Spherox per year. Expressed in a conservative perspective, the potential for more than 115,000 knee-joint treatments per year was calculated in Europe. Marketing approval of Spherox was received in May 2017 from EMA (CODON, 2019). According to the J-TEC company’s IR book, the sales of JACC between FY 2011–2012 were reported to be $10.8 million compared to $371280 at its launch in 2003 (PMDA, 2011). In the field of cardiovascular diseases, according to the FCB Pharmicell Company IR book for Cellgram-AMI, in the South Korea the number of patients with AMI in 2015 was 87,984. The total amount of medical care costs for AMI in the same year was $1749 million (Pharmicell Ltd., 2020). Assessment of Neovasculogen, a product for cardiovascular diseases in Russia, indicated that 5 million people have been diagnosed with PAD. Also, the number of patients with CLI annually amounts up to 145,000, of which around 25% die (Deev et al., 2015b; NOVARTIS, 2019a). Also, the market size for Stempeucel is approximately $1.5 billion worldwide (Lane, 2016). Moreover, the annual reports from Admedus indicated that they achieved revenues of $6.9M million by selling their ADAPT^®^, bio-scaffold tissue technology, realted products (including CardioCel^®^ Neo, VascuCel^®^) in North America, Europe, the MENA region, Asia, Australia, and New Zealand (Cardiocel, 2017). Products based on human cord blood derived HPCs, cover the market of hematologic disorders. Cord blood, as stem cell sources for patients without a donor, has its own market with more than 2000 cord blood hematopoietic stem cell transplants performed each year (WHO, 2013). The other approved product in this field is Zalmoxis. In 2018, Molmed Company projected peak sales of around 100 $ million to be achieved by 2026 for Zalmoxis (Spark Therapeutics Inc., 2019). However, very recently MolMed decided to the withdraw the drug’s conditional marketing authorization (CMA) after Phase III clinical trial results showed that the drug offered no benefit on DFS (Sharma, 2019) and stopped investing in the product. Revenues for Zalmoxis in 2018 is estimated at approximately $4.6 million (MolMed, 2018). In the neurological disorders, by taking into account the production of Nuronata-r in January 2015 and based on sales for June 2015 that were approximately $1.6 million, 2016 sales were estimated to be approximately $4.8 million with a rapid sales growth compared to other stem cell treatments. Also, the domestic patient sales in 2018 were expected to reach near $16.6 million which, together with overseas patients, the final number would be approximately $37.4 million (Commercialized Stem Cell Corestem Inc., 2020). Finally, Spark Therapeutics reported that in the year ended December 31, 2018, they recognized $64.7 million in total revenue, of which $27.0 million was net product sales of LUXTURNA (Spark Therapeutics Inc., 2019).

Overall, also it is worthy to note that according to the Seoane- Vazquez et al. (2019), the treatment cost of Kymriah, sipuleucel-T, Imlygic, and autologous cultured chondrocytes was higher in the United States than in Europe.

## Conclusion and Future Remarks

**FIGURE 3:**
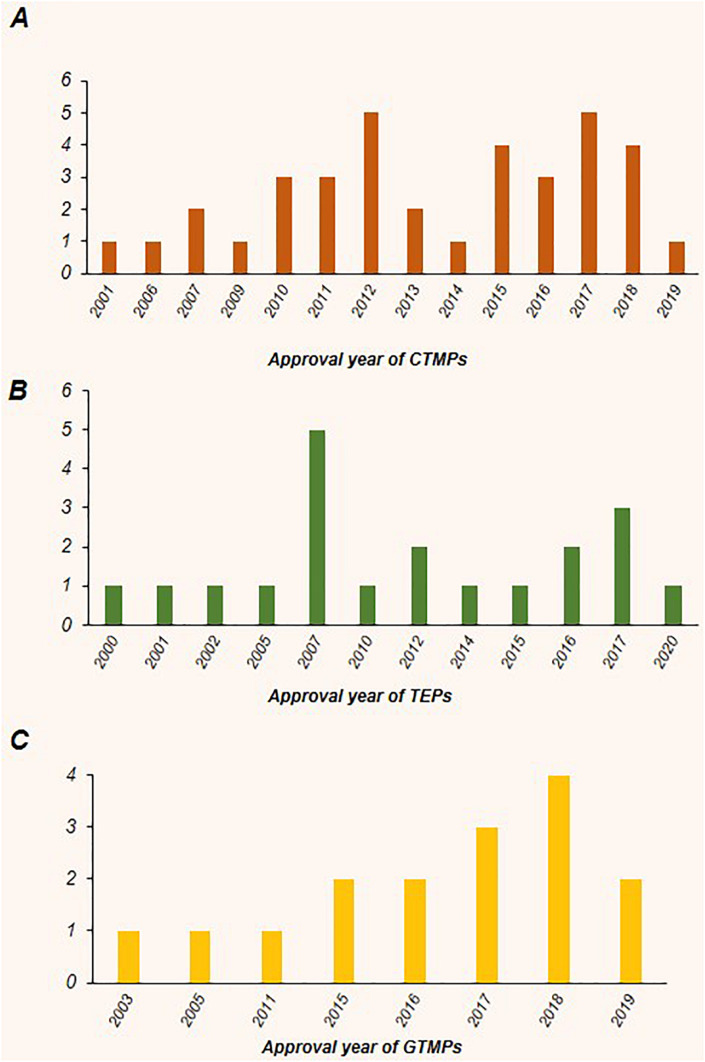
The annual trend of approval for CTMPs, GTMPs, and TEPs worldwide. Most of the products were recently approved. **(A)** In terms of CTMPs, despite a decrease in the number of approved products in 2013 and 2014, a second increased trend can be observed with five products approved in 2017. **(B)** For TEPs, the year 2007 with five approved products showed an increase in the total trend; however, there was a subsequent decrease in the following years. **(C)** There is a noticeable growth trend with respect to gene therapy medicinal products (GTMPs).

Cellular therapies have attracted tremendous attention from numerous researchers, clinicians and from industry, specifically for incurable diseases. As it can be concluded from Tables 1–3, most of the approved products in the recent years belong to the CTMPs, although a decrease in the total amount of approved products occurred in 2013 and 2014, which may be occurred due to financial and regulatory restriction issues. For the next years, there was an increased trend with five products approved in 2017 (Figure 3A). The year 2007 witnessed an increase in the number of approved TEP products; however, in the next years this amount decreased and this field has not experienced any evident growth trend, which can be seen in two other products (Figure 3B). GTMPs have faced an obvious progressive trend regarding the number of approved products and this is clearly apparent from the growth in the trend of these products, which may be due to entrance of big pharma in this field to develop a treatment for refractory conditions or rare diseases which do not have an effective pharmaceutical drug. The latest approved product, Zynteglo, is placed in this class (Figure 3C). It is expected that with the current advances in genetic engineering, products in this field would experience significant growth in following years. The origin of these products is an important subject since the use of autologous, allogeneic or xenogeneic cell sources would have a substantial effect on the financial policies of the companies that produce the regenerative medicine product and the related regulatory agencies. The allogeneic based products have a preferred advantage regarding their capability to be adapted for scale up strategies and also catching large market through massive export. However, as mentioned before, most members of CTMPs and GTMPs have an autologous source, due to the medicinal constraints in regards with immune rejection and the extensive required safety tests for allogeneic cells, while the cells used in tissue engineering based products with local indications, have the higher percentage for allogeneic rather than autologous products and have a greater market and higher sales (Kim et al., 2019). Altogether, it seems that with release of guidelines in different regulatory agencies the regulatory problem could be overcome in next years and trend of allogeneic products would raise noticeably. Market size and price are two main determinants of a product success or failure. Glybera with a both low market size and high price is one example of how these two factors can affect the survival of a certain ATMP in the market. Generally, the average prices for GTMPs are higher in comparison to CTMPs and TEPs. ATMPs related treatments despite their great therapeutic potential are very costly and there are challenges regarding their reimbursement issues. The high price of ATMPs is the result of several factors; high manufacturing costs, complex quality control tests, expensive raw materials, requirement of cold chains for transfer, intellectual property rules, small target populations (in some of cases) and strict regulatory inspections are among the most important reasons.

Due to the significant therapeutic potential of ATMPs for serious conditions in comparison with conventional drugs, as one of the major growth drivers for market, their market share is anticipated to be increasing. Respecting to the new slow shift toward personalized medicine while ATMPs are one of the high potential players in this regard, increasing the value of ATMPs global market size is highly expected. This idea is strongly supported by entrance of big pharma to this field in recent years. Moreover, the market interest has largely affected the number of developed products. This can be seen, for instance, in the percent of approved products for skin related application, as they are placed in the first position of TEPs (60%) and also CTMPs (23.5%).

Overall, the criteria regarding the efficacy of each ATMPs, shows optimistic results and the total numbers of adverse events are not dramatic. Cellular therapy is expected to be a promising area used for treatment of a noticeable quantity of incurable disorders. However, limiting factors regarding the development and uses of ATMPs should be still overcome, including the demand for high-technology systems for cell manufacturing and delivery (reducing production times and costs), vectors for gene modification availability and production, establishment of assays to validate products potency to ultimately improve treatment efficacy while avoiding adverse events and, last but not least the costs of these products and their reimbursability. On the other hand, since regenerative medicine strategies might become a solution for treatment of still incurable diseases and due to massive public and private investments, it is rational to claim that this field has the potential to overcome many of the mentioned limitations in a near future to reach a revolutionary phase in both the pharmaceutical industry and in the clinics.

## Author Contributions

HB, ST, EH-S, and RR conceived the manuscript concept, wrote and final edited the manuscript. All authors participated in the literature search, wrote the manuscript parts, prepared the figures and tables, and read and approved the final manuscript.

## Conflict of Interest

The authors declare that the research was conducted in the absence of any commercial or financial relationships that could be construed as a potential conflict of interest.

## Abbreviations

6MWT: 6-minute walk test
AALS: Appel ALS
AAV: adeno-associated virus
ABPI: ankle brachial pressure index
ACKFBC: Allogeneic Cultured Keratinocytes and Fibroblasts in Bovine Collagen
ADA-SCID: adenosine deaminase severe combined immunodeficiency
ADC: adipose tissue-derived cell
ADL: activities of daily living
AL: allogeneic
ALS: amyotrophic lateral sclerosis
ALSFRS: Amyotrophic Lateral Sclerosis Functional Rating Scale
AMDDC: autologous monocyte-derived mature dendritic cell
AMI: acute myocardial infarction
ASD: atrial septal defect
AT: autologous
ATMP: advanced therapy medicinal product
AT-MSC: adipose tissue-derived mesenchymal stem cell
AVN: avascular necrosis
AVR: aortic valve reconstruction
B-ALL: B-cell precursor acute lymphoblastic leukemia
BCVA: best-corrected visual acuity
BM-MSC: bone marrow-derived mesenchymal stem cell
CAGR: compound annual growth rate
CAR: chimeric antigen receptor
CD: Crohn’s disease
CEA: cultured epidermal autograft
CFDA: China Food and Drug Administration
CLI: critical limb ischemia
COBLT: cord blood transplantation
CR: complete remission
CRT: cardiac resynchronization therapy
*CTLA4Ig*: cytotoxic T-lymphocyte associated protein 4 immunoglobulin
DB: dermal burn
DC: dendritic cell
DCGI: Drug Controller General of India
DDB: deep dermal burn
DFS: disease-free survival
DFU: diabetic foot ulcer
DLBCL: diffuse large B-cell lymphoma
DRR: durable response rate
DTH: delayed type hypersensitivity
EFS: event-free survival
EMA: European Medicines Agency
FDA: Food and Drug Administration
FST: full-field light sensitivity testing
FVC: forced vital capacity
GACI: gel-type autologous chondrocyte implantation
GM-CSF: granulocyte macrophage colony stimulating factor
GTMP: Gene Therapy Medicinal Product
GvHD: graft-versus-host disease
HAS: Heath Administration of Singapore
HCEpC: human corneal epithelial cell
HDFn: human dermal fibroblasts, neonatal
HEKn: human epidermal keratinocytes, neonatal
HPC: hematopoietic progenitor cell
HNSCC: head and neck squamous cell carcinoma
*hRPE65*: human retinal pigment epithelium 65 kDa
HSCT: hematopoietic stem cell transplantation
HSV: herpes simplex virus
ICRS: International Cartilage Repair Society
IFN-γ: interferon gamma
IKDC: International Knee Documentation Committee
irRC: immune-related response criteria
KLH: keyhole limpet hemocyanin
KOOS: knee injury and osteoarthritis outcome score
KSS: Knee Society Scoring system
LCC: living cellular construct
LPLD: lipoprotein lipase deficiency
LVEDV: left ventricular end-diastolic volume
LVEF: left ventricular ejection fraction
LVESV: end-systolic volume
LVRR: left ventricular reverse remodeling
mCRPC: metastatic castration-resistant prostate cancer
MFDS: Ministry of Food and Drug Safety
MLMT: multi-luminance mobility test
MOCART: Magnetic Resonance Observation of Cartilage Repair Tissue
MOH: Ministry of Health
mPP: modified per protocol
MSC: mesenchymal stem cell
MSS: MRI Scoring of Severity
NGFR: nerve growth factor receptor
NLF: nasolabilal fold
NRM: non-relapse mortality
NSCLC: non-small cell lung carcinoma
OA: osteoarthritis
OCD: osteochondritis dissecans
OR: overall response
ORR: objective response rate
ORR: overall response rate
ORR: overall remission rate
OS: overall survival
PAD: peripheral artery disease
PAP: prostatic acid phosphatase
PBMNC: peripheral-blood mononuclear cell
Pca: prostate cancer
PCI: percutaneous coronary intervention
PFU: plaque forming unit
PMBCL: primary mediastinal large B-cell lymphoma
PMDA: Pharmaceuticals and Medical Devices Agency
PR: partial remission
PRP: platelet-rich plasma
PWD: pain-free walking distance
QOL: quality of life
r/r: relapsed or refractory
RECIST: Response Evaluation Criteria in Solid Tumor
RFS: recurrence free survival
RFS: relapse-free survival
SCTMP: somatic cell therapy medicinal product
SDCS: skeletal myoblast-derived cell sheet
SOC: standard of care
SPECT: single-photon emission computed tomography
SRA: sports and recreational activity
TBSA: total body surface area
TEAEs: treatment emergent adverse events
TEP: tissue engineered product
TGA: Therapeutic Goods Administration
TL-pulsed DC: tumor lysate-pulsed dendritic cell
TNC: total nucleated cell
TREC: T-cell receptor excision circle
TTF: time to treatment failure
TTP: time to progression
UCCBB: University of Colorado Cord Blood Bank
SMA: spinal muscular atrophy
SMN: survival motor neuron
VAS: visual analog scale
VEGF: vascular endothelial growth factor
VG: vector genome
VLU: venous leg ulcer
VP: viral particle
VSD: ventricular septal defect
Xn: xenogeneic.
